# Sintilimab plus anlotinib as second‐ or third‐line therapy in metastatic non‐small cell lung cancer with uncommon epidermal growth factor receptor mutations: A prospective, single‐arm, phase II trial

**DOI:** 10.1002/cam4.6548

**Published:** 2023-09-18

**Authors:** Kaiyan Chen, Yanjun Xu, Zhiyu Huang, Xiaoqing Yu, Wei Hong, Hui Li, Xiaoling Xu, Hongyang Lu, Fajun Xie, Jun Chen, Youzu Xu, Yun Fan

**Affiliations:** ^1^ Zhejiang Cancer Hospital, Hangzhou Institute of Medicine (HIM) Chinese Academy of Sciences Hangzhou China; ^2^ Department of Thoracic Medical Oncology Zhejiang Cancer Hospital Hangzhou China; ^3^ Department of Clinical Trial Zhejiang Cancer Hospital Hangzhou China; ^4^ Department of Radiotherapy and Chemotherapy The Affiliated People's Hospital of Ningbo University Ningbo China; ^5^ Department of Respiratory and Critical Care Medicine Taizhou Hospital of Zhejiang Province Affiliated to Wenzhou Medical University Taizhou China

**Keywords:** anlotinib, anti‐angiogenic, non‐small cell lung cancer (NSCLC), PD‐1, sintilimab, uncommon *EGFR* mutations

## Abstract

**Background:**

Patients with non‐small‐cell lung cancer (NSCLC) and uncommon *EGFR* alterations typically have worse treatment outcomes than patients with classically *EGFR*‐mutated NSCLC. This study aimed to investigate the efficacy and safety of PD‐1 blockade with sintilimab plus anti‐angiogenic treatment with anlotinib in patients with NSCLC harboring uncommon *EGFR* mutations.

**Methods:**

Patients with metastatic NSCLC harboring uncommon *EGFR* mutations after two previous treatments, including a platinum‐based chemotherapy regimen and a targeted treatment (or chemotherapy only for patients harboring *EGFR* ex20ins), received sintilimab combined with anlotinib. The primary endpoint was objective response rate (ORR).

**Results:**

At data cutoff (September 27, 2022), median follow‐up was 22.3 months (range, 1.2–37.6). Among 21 enrolled patients, 12 had *EGFR* ex20ins and nine had other uncommon *EGFR* mutations such as L861Q, G719A, and G709X. Overall, eight patients (38.1%) achieved an objective response, and 18 (85.7%) achieved disease control. Median (95% CI) progression‐free survival (PFS) was 7.0 (5.4–8.6) months, and median overall survival (OS) was 20.0 (15.6–24.4) months. The 12‐month PFS rate (95% CI) was 22.2% (7.4–42.0), and the 12‐month OS rate was 66.7% (42.5–82.5). Patients harboring *EGFR* ex20ins had similar ORR and PFS to those with other mutations. Six patients (28.6%) experienced grade 3 treatment‐related adverse events (TRAEs); hand‐foot syndrome was the most common grade 3 TRAE (2 patients; 9.5%). No grade ≥4 TRAEs were observed.

**Conclusions:**

The combination of sintilimab and anlotinib demonstrated durable efficacy and was generally well tolerated in patients with NSCLC and uncommon *EGFR* mutations who had received prior standard‐of‐care treatments. (ClinicalTrials.gov identifier: NCT04790409).

## 1 BACKGROUND

Lung cancer is the leading cause of cancer‐related death globally.^1^ Epidermal growth factor receptor (*EGFR*) gene mutations are the most prevalent oncogenic driver alteration among Asian patients with lung adenocarcinoma and may be present in up to 51.4% of patients.^2^ Approximately 90% of *EGFR* mutations in non‐small cell lung cancer (NSCLC) are “classic mutations” (19del, L858R, etc.) that are sensitive to treatment with EGFR tyrosine kinase inhibitors (TKIs).^3^, ^4^ Indeed, EGFR TKIs are the recommended first‐line treatment for *EGFR*‐mutated NSCLC, with an associated median progression‐free survival (PFS) of 9.0–18.9 months.^5^, ^6^, ^7^, ^8^, ^9^ The other 10% of *EGFR* mutations in NSCLC comprise rare mutations such as *EGFR* ex20ins, G719X, L861Q, and S768I/V, which confer inferior responses to EGFR TKI treatment.^2^

The EGFR TKIs afatinib and osimertinib are recommended for the treatment of NSCLC harboring G719X, S768I, and L861Q mutations. These agents have demonstrated moderate anti‐tumor activity, with a median PFS of 8.2–10.7 months.^10^, ^11^ However, when TKI resistance occurs, the only remaining treatment option is platinum‐based chemotherapy, which is associated with a median PFS of only 4–5 months.^12^ In addition, patients with less common *EGFR* mutations typically achieve a poor response to EGFR TKIs. For example, the ex20ins mutation has long been considered an “undruggable target” and is associated with a worse median overall survival (OS) than other uncommon *EGFR* point mutations.^10^, ^13^ In one study of patients with ex20ins treated with afatinib, the objective response rate (ORR) was only 8.7%, and the median PFS was 2.7 months,^10^ suggesting primary resistance to first‐ or second‐generation EGFR TKIs may be common. To date, amivantamab and mobocertinib (TAK‐788) are approved for the second‐line treatment of patients with NSCLC and ex20ins, with ORRs of 40%,^14^ and 28%,^15^ respectively. However, as these drugs are not available in many countries, the treatment of patients with ex20ins is modeled on that used for patients with oncogene‐negative NSCLC (e.g., salvage chemotherapy). After progression on first‐line chemotherapy, second‐line docetaxel has limited therapeutic effect and substantial toxicity, with a median OS of only 6 months.^16^ Collectively, the limited accessibility and efficacy of targeted therapies for patients with uncommon *EGFR* mutations (especially ex20ins) mean that new treatment strategies to improve clinical outcomes are urgently needed.

Immune checkpoint inhibitors (ICIs) targeting the programmed death 1 (PD‐1)/PD ligand 1 (PD‐L1) pathway have revolutionized the treatment of advanced NSCLC; however, the observed OS benefit with second‐line ICI monotherapy over docetaxel in patients with NSCLC has failed to extend to *EGFR*‐mutated patients.^17^, ^18^ Retrospective studies have suggested that some *EGFR* mutation subtypes may be associated with sensitivity to ICIs; specifically, patients with ex20ins or G719X appear to achieve a longer PFS and a higher ORR than patients with classic *EGFR* mutations.^19^, ^20^

Combining ICIs with antiangiogenic agents can result in synergistic antitumor effects.^21^ For example, in the IMpower150 trial, the addition of atezolizumab to bevacizumab and chemotherapy in patients with EGFR‐TKI drug‐resistant NSCLC led to improved efficacy.^22^ The Orient‐31 study reported an advantage of a chemotherapy‐containing four‐drug therapy (sintilimab plus bevacizumab biosimilar plus cisplatin and pemetrexed) over chemotherapy alone in patients with *EGFR*‐mutated NSCLC after TKI failure.^23^ However, as these two trials included few patients with non‐classic *EGFR* mutations (26.0% and 6.3%, respectively) and reported a relatively high proportion of patients experiencing grade 3 or 4 treatment‐related adverse events (TRAEs) with the four‐drug regimen, it is necessary to identify regimens with better tolerability and comparable efficacy for patients with NSCLC harboring rare *EGFR* mutations.

Sintilimab is a novel anti–PD‐1 monoclonal antibody (mAb) with encouraging antitumor activity in advanced NSCLC.^24^ The combination of sintilimab with chemotherapy as first‐line therapy for advanced NSCLC has been shown to improve ORR and PFS.^25^, ^26^ Anlotinib is an anti‐angiogenic TKI that targets VEGFR 1/2/3, FGFR1‐4, PDGFR a/b, and c‐Kit and has been approved as a third‐line treatment for NSCLC in China.^27^ Sintilimab plus anlotinib has shown improved efficacy compared with chemotherapy alone in first‐line for patients with advanced NSCLC.^28^ In order to reduce the toxicity associated with the four‐drug regimen and treat NSCLC with rare *EGFR* mutations after TKI failure, sintilimab plus anlotinib represents a promising treatment choice after failure of standard of care treatment. In this prospective phase 2 study, we investigated the efficacy and safety of sintilimab plus anlotinib without chemotherapy in pretreated patients with advanced NSCLC harboring uncommon *EGFR* mutations.

## 2 METHODS

### 2.1 Study design and participants

This was a single‐center, open‐label, phase II trial conducted at Zhejiang Cancer Hospital, China (ClinicalTrials.gov identifier: NCT04790409). The study was conducted following the provisions of the Declaration of Helsinki and Good Clinical Practice guidelines. The study protocol was approved by the Ethics Commission of Zhejiang Cancer Hospital (IRB‐2019‐81) and written informed consent was provided by all patients before inclusion.

Eligible patients were 18–70 years old and had histologically or cytologically confirmed treatment‐experienced advanced NSCLC (AJCC 8th edition), measurable disease, and disease progression after at least two treatment regimens that included one platinum‐containing chemotherapy and one EGFR TKI. Patients with *EGFR* ex20ins mutations who had experienced disease progression after only platinum‐based chemotherapy were also eligible. Other key eligibility criteria included an Eastern Cooperative Oncology Group (ECOG) performance status of 0 or 1 and adequate organ function. Key exclusion criteria included components of small cell carcinoma, symptomatic central nervous system metastases, central squamous cell carcinoma with a cavity, active hemorrhage or risk of hemorrhage, and prior immunotherapy.

### 2.2 Systemic therapy

Eligible patients received sintilimab (Innovent [Suzhou] Biopharmaceutical Co., Ltd.) 200 mg intravenously once on Day 1 and subsequently every 3 weeks, plus anlotinib (Chia Tai Tianqing Pharmaceutical Group Co., Ltd.) 12 mg orally on Days 1–14 of the 21‐day treatment cycle, until disease progression or death, unacceptable toxicity, withdrawal of consent, or for a maximum of 24 months (35 cycles) of sintilimab. No other antitumor therapy was allowed before disease progression. For sintilimab, no dose adjustment was allowed. For anlotinib, the investigator could reduce the dose to 10 mg, 8 mg, or permanently discontinue treatment according to drug‐related toxicity. Patients with intolerable adverse events (AEs) that led to a delay or discontinuation of one study drug continued treatment with the other drug.

### 2.3 Outcomes and measurements

The primary endpoint was ORR, assessed by the investigator according to Response Evaluation Criteria in Solid Tumors (RECIST) v1.1. Secondary endpoints were PFS, OS, disease control rate (DCR, defined as the best overall response [BOR] of stable disease [SD] or better), duration of response (DOR, defined as the time from first documented complete or partial response [CR or PR] to disease progression or death), and safety and tolerability. Tumor responses were measured using computed tomography or magnetic resonance imaging. Patients underwent imaging evaluations at baseline (treatment initiation), Week 6, Week 12, and then every 9 weeks. After Week 48, imaging evaluations were performed every 12 weeks. Imaging evaluations could be performed within ±7 days of the scheduled visit. CR and PR were confirmed radiologically at least 4 weeks after the first response. Safety was recorded from enrollment until 90 days after the last dose of study treatment. Treatment‐emergent adverse events (TEAEs), treatment‐related adverse events (TRAEs), and laboratory biochemical data parameters were summarized using the Common Terminology Criteria for Adverse Events (CTCAE), version 5.01.

### 2.4 Biomarker analysis

Before treatment initiation, tumor tissue samples from initial diagnosis or post‐resistance biopsy were collected for evaluation of PD‐L1, CD4, CD8, CD163, Foxp3, and LAG3 expression in the tumor microenvironment. PD‐L1 expression was measured by a 22C3 pharmDx assay (Agilent Technologies), and PD‐L1 positivity (PD‐L1+) was defined as a PD‐L1 tumor proportion score (TPS) ≥1%. CD4, CD8, CD163, Foxp3, and LAG3 staining on immune cells was reported as the proportion of positive cells among all nucleated cells in the stromal compartments, and scoring was recorded as negative (<10%) or positive (≥10%).

Epidermal growth factor receptor mutations were assessed by using amplification refractory mutation system polymerase chain reaction (ARMS‐PCR; Amoy Diagnostics) or next‐generation sequencing (NGS, Burning Rock Biotech and Nanjing Geneseeq Technology Inc.). Among them, 12 patients were detected by ARMS‐PCR and nine patients by NGS. The details of targeted NGS data are described in the Appendix S1. The sequencing coverage and quality statistics for each sample are summarized in Table S1.

### 2.5 Statistical analysis

The sample size was calculated using a Simon's 2‐stage design^29^ with alpha set at 0.1, power set to 0.83 and assuming an ORR of 10% for historical treatment (single‐drug docetaxel monotherapy in second line), and 30% for sintilimab plus anlotinib. Consequently, it was required that 14 eligible patients receive treatment in the first stage of the study with at least one response to continue the enrollment. In stage 2, four additional patients would be enrolled for a total target enrollment of 18. Overall, if four responses or more were observed, the treatment regimen would be considered successful. Allowing for an estimated drop‐out rate of 15%, a sample size of 21 patients was planned.

The main analysis included all patients who met the inclusion criteria and received at least one dose of sintilimab combined with anlotinib (intent‐to‐treat [ITT] population). The safety analysis was also performed in the ITT population. ORR and DCR were analyzed using the Clopper–Pearson method. PFS, OS, and DOR were estimated using the Kaplan–Meier method. Baseline demographics and safety data were summarized using descriptive statistics. Cox proportional‐hazards regression models were used for a univariate analysis to identify potential factors associated with clinical outcomes. All statistical tests were two‐sided, and *p* < 0.05 was considered statistically significant. All statistical analyses were performed using R software (version 3.4.3).

## 3 RESULTS

### 3.1 Patients

Between August 2019 and December 2021, a total of 21 patients were enrolled. Baseline characteristics of the enrolled patients are shown in Table 1. Overall, 66.7% were male, the median age was 61 years, 90.5% had lesions classified histologically as adenocarcinoma, the majority (66.7%) of patients had an ECOG performance status of 1, and 61.9% had never smoked. Prior to enrollment, 15 patients (71.4%) had received ≥2 treatments. Preexisting brain or liver metastases were present in 9 (42.9%) and 4 (19.0%) patients, respectively. Twelve patients (57.1%) had confirmed ex20ins and nine (42.9%) had G719A, L861Q, or G709X *EGFR* mutations. Seven patients (33.3%) had detectable intra‐tumoral expression of PD‐L1 (TPS ≥1%), while 14 patients (66.7%) had a PD‐L1 TPS that was missing or <1%. Among the 15 patients with evaluable samples, positive expression of CD4+, CD8+, Foxp3+, CD163+, and LAG3+ immune cells was identified in 53.3% (8/15), 33.3% (5/15), 60.0% (9/15), 40.0% (6/15), and 20.0% (3/15), respectively. Dual‐positivity for expression of PD‐L1 and CD8 was identified in 20.0% (3/15) of patients.

**TABLE 1:** Patient demographics and baseline characteristics.

Characteristic	Patients (*N* = 21)
Median (range) age, years	61 (36–71)
Sex	
Male	14 (66.7)
Female	7 (33.3)
ECOG performance status	
0	7 (33.3)
1	14 (66.7)
Histology	
Adenocarcinoma	19 (90.5)
Non‐adenocarcinoma	2 (9.5)
Smoking history	
Never	13 (61.9)
Current/previous	8 (38.1)
Family history of NSCLC	
No	14 (66.7)
Yes	7 (33.3)
Uncommon mutation type	
G719X, etc.	9 (42.9)
ex20ins	12 (57.1)
Brain metastases	9 (42.9)
Liver metastases	4 (19.0)
Prior lines of treatment	
1	6 (28.6)
2	11 (52.4)
≥3	4 (19.0)
PD‐L1 TPS, %	
<1	8 (38.1)
1–49	4 (19.0)
>50	3 (14.3)
Missing	6 (28.6)

*Note*: Data are *n* (%) unless otherwise stated.

Abbreviations: ECOG, Eastern Cooperative Oncology Group; NSCLC, non‐small cell lung cancer; TPS, tumor proportion score.

At data cutoff (September 27, 2022), the median duration of follow‐up was 22.3 months (range: 1.2–37.6), at which time 10 patients were still alive (three still on treatment), and 11 had died.

### 3.2 Efficacy

**FIGURE 1:**
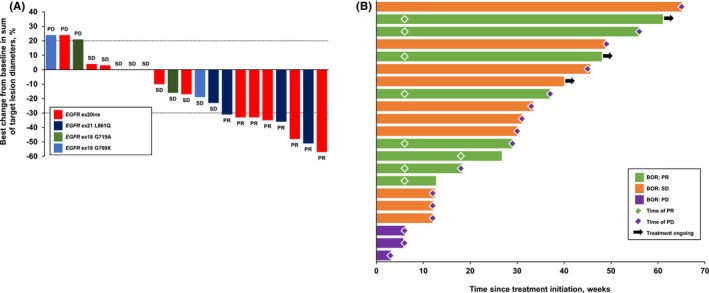
(A) Best change from baseline in sum of target lesion diameters, according to type of *EGFR* mutation. Column labels indicate BOR. BOR, best overall response; ex, exon; ins, insertion; PD, progressive disease; PR, partial response; SD, stable disease; (B) Duration of treatment and time to response. BOR, best overall response; PD, progressive disease; PR, partial response; SD, stable disease.

**FIGURE 2:**
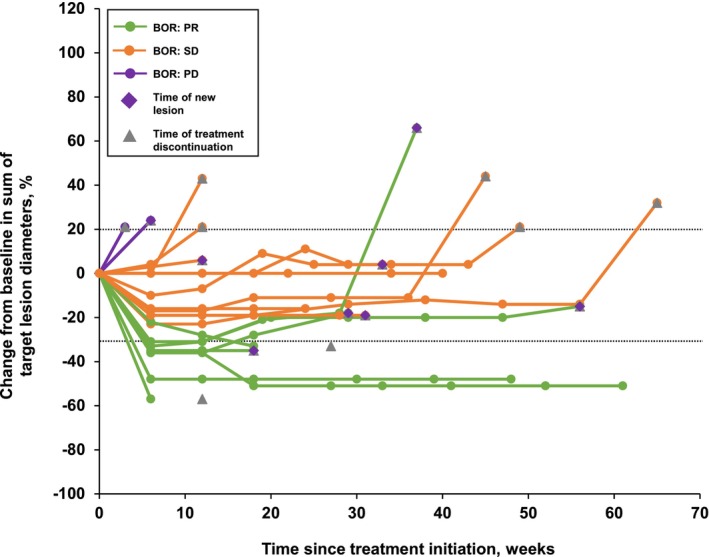
Change from baseline in sum of target lesion diameters over time. Three patients had a BOR of PD; two patients had a change from baseline in target lesion diameters of 24% over 6 weeks, so these data points overlap on the plot. BOR, best overall response; PD, progressive disease; PR, partial response; SD, stable disease.

In the ITT population, the majority of patients (61.9%) had a reduction in tumor burden (Figure 1A). Responses were observed as early as 6 weeks (Figure 1B), with eight patients (38.1%) achieving an objective response and 18 (85.7%) with disease control (Table S2). The median DOR was 5.2 months (95% CI: 0.0–11.2). Overall, SD and/or a PR were noted in most patients during the first 6–7 months of combined sintilimab and anlotinib treatment (Figure 2). The ORR was consistent across different *EGFR* mutation patterns and the presence or absence of liver or brain metastases (*EGFR* ex20ins, 41.7% vs other mutations, 33.3%; liver metastasis or not: 25.0% vs 41.2%; brain metastasis or not: 33.3% vs 41.7%; all *p* > 0.05, Table S2).

**FIGURE 3:**
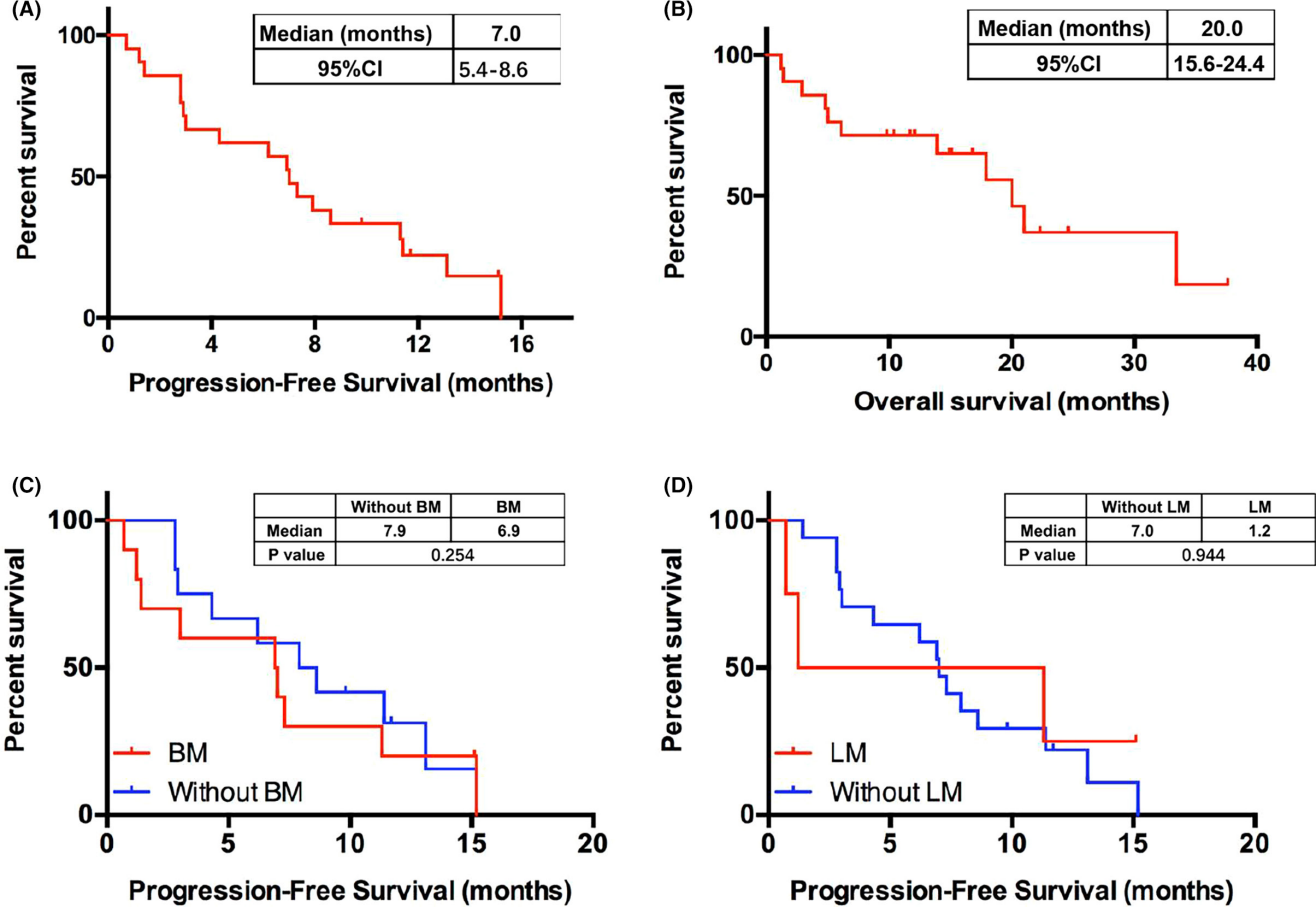
Progression‐free survival (PFS) overall and overall survival (OS) overall and in subgroups of interest. Kaplan–Meier plots indicating: (A) PFS among all patients; (B) OS among all patients; (C) PFS for patients with (red trace) or without (blue trace) brain metastasis; (D), PFS for patients with (red trace) or without (blue trace) liver metastasis.

The median PFS was 7.0 months (95% CI, 5.4–8.6), with a 6‐month PFS rate of 57.1% (95% CI, 33.8–74.9) and a 12‐month PFS rate of 22.2% (95% CI, 7.4–42.0; Figure 3A). The median OS was 20.0 months (95% CI, 15.6–24.4), with a 6‐month OS rate of 71.4% (95% CI, 47.2–86.0) and a 12‐month OS rate of 66.7% (95% CI, 42.5–82.5; Figure 3B). Patients carrying *EGFR* ex20ins had a similar PFS to patients with other mutation patterns (4.3 vs 7.3 months, *p* = 0.685). Moreover, patients with brain metastases had a similar PFS to those without brain metastases (6.9 vs 7.9 months, *p* = 0.254; Figure 3C). Although patients with liver metastases had worse PFS than those without liver metastases, the difference was not statistically significant (1.2 vs 7.0 months, *p* = 0.944; Figure 3D).

**FIGURE 4:**
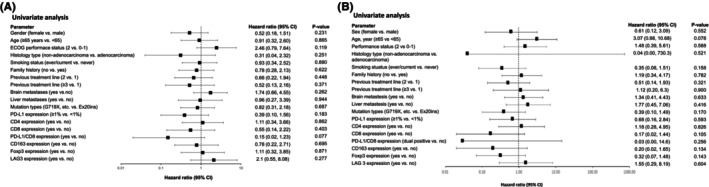
(A) Univariate analysis of progression‐free survival according to baseline patient characteristics; (B) Univariate analysis of overall survival according to baseline patient characteristics.

A univariate analyses showed no statistically significant association between PFS and PD‐L1 expression or the presence of CD4+, CD8+, Foxp3+, CD163+, and LAG3+ immune cells (Figure 4A). There was a trend for patients who were double positive for PD‐L1 and CD8 to have a lower risk for disease progression than those who were not double positive (HR, 0.15 [95% CI, 0.02–1.23]; *p* = 0.077; Figure 4A) but this result did not reach statistical significance. No factors were observed to be associated with OS (Figure 4B).

### 3.3 Safety

The median duration of treatment was 7.0 months (range, 0.7–15.2) for combination therapy. At least one TRAE of any grade occurred in 20 patients (95.2%), the most common of which were hypothyroidism (eight patients, 38.1%), increased aspartate aminotransferase (AST; seven patients, 33.3%) and proteinuria (seven patients, 33.3%; Table 2). Grade 3 TRAEs occurred in six patients (28.6%). The most common grade 3 TRAEs were hand‐foot syndrome (two patients, 9.5%), hypertension (one patient, 4.8%), cerebral infarction (one patient, 4.8%), fatigue (one patient, 4.8%), and immune‐related pneumonitis (one patient, 4.8%; Table 2). Four patients (19.0%) required a dose reduction of anlotinib owing to unacceptable toxicity. Two patients (9.5%) discontinued treatment because of TRAEs (one experienced pneumonitis [anlotinib continued], and one experienced cerebral infarction [discontinued both drugs]). There were no grade 4–5 TRAEs and no new safety signals were observed.

**TABLE 2:** Summary of treatment‐related adverse events (intent‐to‐treat population; *N* = 21).

TRAE	Any grade	Grade 3
Hypothyroidism	8 (38.1)	0
Increased AST	7 (33.3)	0
Proteinuria	7 (33.3)	0
Fatigue	6 (28.6)	1 (4.8)
Increased ALT	6 (28.6)	0
Thrombocytopenia	6 (28.6)	0
Hand‐foot syndrome	5 (23.8)	2 (9.5)
Hyperthyroidism	4 (19.0)	0
Rash	4 (19.0)	0
Hypertension	3 (14.3)	1 (4.8)
Anemia	2 (9.5)	0
Diarrhea	2 (9.5)	0
Hypertriglyceridemia	2 (9.5)	0
Hypoalbuminemia	2 (9.5)	0
Hyponatremia	2 (9.5)	0
Immune‐related pneumonitis	2 (9.5)	1 (4.8)
Increased creatinine	2 (9.5)	0
Increased uric acid	2 (9.5)	0
Neutropenia	2 (9.5)	0
Cerebral infarction	1 (4.8)	1 (4.8)
Epistaxis	1 (4.8)	0
Hoarseness	1 (4.8)	0
Hypercholesterolemia	1 (4.8)	0
Increased direct bilirubin	1 (4.8)	0
Oral ulcer	1 (4.8)	0
Nausea	1 (4.8)	0
Vomiting	1 (4.8)	0

*Note*: Data are *n* (%).

Abbreviations: ALT, alanine aminotransferase; AST, aspartate aminotransferase; TRAE, treatment‐related adverse event.

## 4 DISCUSSION

To our knowledge, this is the first study to report the efficacy and safety of sintilimab plus anlotinib in pretreated patients with advanced NSCLC harboring uncommon *EGFR* mutations. Our study revealed that this combined therapy has favorable efficacy, durability, and tolerability. Interestingly, favorable clinical outcomes were observed for patients with PD‐L1 and CD8 dual positivity. Based on our findings, sintilimab plus anlotinib has promise as a chemotherapy‐free treatment regimen for patients with NSCLC harboring uncommon *EGFR* mutations after disease progression on standard of care treatment.

Previous studies in NSCLC have revealed the effectiveness of combined treatment with ICIs and antiangiogenic therapies. The inhibition of angiogenesis potentiates PD‐1/PD‐L1 blockade by reducing hypoxia, promoting the infiltration of CD8 T cells, and inhibiting recruitment of tumor‐associated macrophages.^30^ In the phase 1 JVDF trial, a combination of the anti‐VEGFR‐2 antibody, ramucirumab, plus the anti‐PD‐1 antibody pembrolizumab led to an ORR of 42.3%, including patients previously treated for NSCLC.^31^ A phase 1b study of first‐line sintilimab plus anlotinib in patients with advanced NSCLC reported a high ORR (72.7%) and DCR (100%),^32^ leading to an ongoing phase 2 randomized study of sintilimab plus anlotinib versus chemotherapy that has so far reported an ORR of 50% (chemotherapy: 33%) and a median PFS of 10.8 months (chemotherapy: 5.7 months; HR = 0.4, *p* = 0.002).^28^ In addition, second‐line therapy with the anti‐PD‐1 mAb camrelizumab and the VEGFR‐2 TKI apatinib in patients with advanced nonsquamous NSCLC was associated with an ORR of 30.9% and a median PFS of 5.7 months.^33^

In the current study, 38.1% of patients who received late‐line treatment with sintilimab plus anlotinib achieved an objective response, and 85.7% achieved disease control. Overall, the median PFS was 7.0 months, and median OS was 20.0 months. Interestingly, given the diverse clinical settings, the ORR for sintilimab plus anlotinib observed in the present study (38.1%) compares favorably with previous studies of anlotinib monotherapy (9.2%)^27^ and sintilimab monotherapy (25.5%) in NSCLC.^34^ A retrospective study of 28 patients harboring *EGFR* ex20ins who received a PD‐1/PD‐L1 inhibitor (with or without a CTLA‐4 inhibitor), reported an ORR of 10.7% (3/28) and a median PFS of 1.9 months^19^; seven patients with *EGFR* G719X who received PD‐1 inhibitor monotherapy had an ORR of 28.6% and a PFS of 4.8 months.^19^ However, cabozantinib plus atezolizumab only demonstrated modest activity in 30 patients with previously‐treated advanced *EGFR*‐mutated NSCLC (ORR = 7%, DCR = 63%, median PFS = 2.7 months, and median OS = 6.1 months).^35^ In contrast, anlotinib combined with ICIs exhibited a longer median PFS (4.3 months vs 3.6 months, *p* = 0.005), and OS (14.2 months vs 9.0 months *p* = 0.029) than chemotherapy for the late‐line treatment of patients with NSCLC and EGFR‐TKI resistance.^36^ Additionally, the phase 3 Orient‐31 trial reported that sintilimab plus the bevacizumab biosimilar IBI305 and chemotherapy led to significant improvements in PFS over chemotherapy alone in patients with advanced *EGFR*‐mutated NSCLC after TKI failure (6.9 vs 4.3 months; *p* < 0.001).^37^ ORR was also higher in the four‐drug group versus chemotherapy (44% vs 25%).^37^ Taken together, these data show that sintilimab plus anlotinib has exhibited better anti‐tumor activity than other similar combinations of a multi‐target angiogenesis inhibitor and ICI and shown comparable efficacy to a four‐drug regimen in patients with pre‐treated NSCLC with uncommon *EGFR* mutations.

While anti‐tumor activity was observed across multiple subgroups in this study, we noted a trend towards higher ORR for patients harboring *EGFR* ex20ins versus other *EGFR* tumor mutations (41.7% vs 33.3%). However, the statistical analysis was limited by the small sample size. The development of selective TKIs (e.g., mobocertinib, sunvozertinib, and CLN‐081) and a bi‐specific antibody (amivantamab) represent major advances for patients with this hard‐to‐treat molecular alteration. However, ORRs and median PFS values are yet to exceed 41% and 12 months, respectively.^38^, ^39^, ^40^ Therefore, new treatment strategies, especially those containing ICIs, are necessary in patients with NSCLC harboring *EGFR* ex20ins. In this regard, the combination of sintilimab and anlotinib is a promising treatment regimen in this patient population.

Among the nine patients with brain metastases at baseline, those with brain metastases had a similar overall PFS to those without brain metastases. Although only two patients had evaluable brain lesions, one achieved intracranial PR and one achieved intracranial SD. Both patients sustained intracranial benefit when extracranial lesions showed progression. The intracranial responses observed were active, although this requires further validation in larger patient cohorts.

In this study, we evaluated the association between survival outcomes and tumor PD‐L1 expression and immune‐cell infiltration. Our results suggest that PFS and OS following treatment with sintilimab combined with anlotinib are not affected by PD‐L1 status, in line with prior studies of ICIs combined with antiangiogenic agents in patients with metastatic NSCLC.^30^, ^32^ PD‐L1 expression can be induced by genetic alterations and inflammatory cytokines, such as interferon (IFN)‐γ. However, in *EGFR*‐mutated tumors, PD‐L1 expression may only reflect EGFR signaling rather than effector T‐cell activity,^41^, ^42^ abrogating the predictive utility of PD‐L1 expression in *EGFR*‐mutated NSCLC. In the present study, the efficacy of combination therapy was similar between patients with different abundances of immune cells (CD4+, CD8+, Foxp3+, CD163+, and LAG3+). It is possible that these single biomarkers do not comprehensively reflect the characteristics of the tumor microenvironment in *EGFR*‐mutated NSCLC, resulting in poor predictive performance.^43^ Additionally, consistent with our preclinical research,^44^ patients with dual positivity for PD‐L1 and CD8 TILs showed a trend toward a lower risk of disease progression versus those without dual positivity (HR, 0.15 [95% CI, 0.02–1.23]), although this result did not reach statistical significance and the statistical analysis was limited by the small sample size. To date, data on the mechanism and predictive role of PD‐L1 expression and immune‐cell infiltration in NSCLC with uncommon *EGFR* mutations are scarce, and further research is needed to inform patient stratification based on these biomarkers.

Combined treatment with sintilimab plus anlotinib was generally well tolerated, and the safety profile was consistent with that of either drug as monotherapy.^27^, ^45^ The incidence of severe grade 3 TRAEs (28.6%) was relatively low, and there were few treatment discontinuations due to TRAEs. Hand‐foot syndrome was the most common grade ≥3 TRAE (9.5%), which is consistent with the known side effects of angiogenesis inhibitors.^46^ No grade 4 or 5 TRAEs were observed.

Limitations of this study include its single‐arm design with a relatively small sample size and a potential for selection bias due to the lack of a control group. In addition, immunohistochemistry results could not be obtained from every patient due to sample accessibility, and there were variable tumor tissue resources. Therefore, the observed trend toward better response among patients with dual expression of PD‐L1/CD8 requires further prospective research for verification.

## 5 CONCLUSIONS

This phase 2 study demonstrated that the combination of sintilimab and anlotinib has promising anti‐tumor activity and is generally well tolerated in patients with advanced, pre‐treated NSCLC harboring uncommon *EGFR* mutations. Further evaluation of this treatment strategy in a well‐designed, prospective trial with a larger cohort is warranted.

## AUTHOR CONTRIBUTIONS

**Kaiyan Chen:** Conceptualization (lead); funding acquisition (supporting); writing – original draft (lead). **Yanjun Xu:** Data curation (equal); formal analysis (equal). **Zhiyu Huang:** Data curation (equal). **Xiaoqing Yu:** Data curation (equal). **Wei Hong:** Data curation (equal). **Hui Li:** Formal analysis (equal). **Xiaoling Xu:** Formal analysis (equal). **Hongyang Lu:** Data curation (equal). **Fajun Xie:** Formal analysis (equal). **Jun Chen:** Formal analysis (equal). **Youzu Xu:** Formal analysis (equal). **Yun Fan:** Funding acquisition (lead); project administration (lead); supervision (lead); writing – review and editing (lead).

## FUNDING INFORMATION

This work was supported by Beijing Xisike Clinical Oncology Research Foundation (Grant Y‐XD2019‐052), Medical Health Science and Technology Project of Zhejiang Province (Grant 2023KY601), and National Natural Science Foundation of China (Grant 81972718).

## CONFLICT OF INTEREST STATEMENT

None of the authors has a conflict of interest related to the study.

## ETHICS STATEMENT

This study conformed to the provisions of the Declaration of Helsinki and Good Clinical Practice guidelines. The study protocol was approved by the Ethics Commission of Zhejiang Cancer Hospital (IRB‐2019‐81) and written informed consent was provided by all patients before inclusion.

## Supporting information


Appendix S1.

## Data Availability

The data underlying this article will be shared on reasonable request to the corresponding author.
