# Supplementary material for: Sintilimab plus anlotinib as second‐ or third‐line therapy in metastatic non‐small cell lung cancer with uncommon epidermal growth factor receptor mutations: A prospective, single‐arm, phase II trial
Source: Cancer Med. 2023 Sep 18;12(19):19460–70. doi: 10.1002/cam4.6548 (PMC10587987; doi:10.1002/cam4.6548)
Supplement: Supplementary file 1 — Appendix S1. [file CAM4-12-19460-s001.docx]

**(Supplementary) Materials and Methods**

***DNA isolation and capture-based targeted DNA sequencing***

DNA isolation and targeted sequencing were performed in Burning Rock Biotech (Guangzhou, China) and Nanjing Geneseeq Technology Inc. (Nanjing, China), two commercial clinical laboratories in China. Briefly, tissue DNA was extracted from formalin-fixed, paraffin-embedded (FFPE) tumor tissues using QIAamp DNA FFPE tissue kit (Qiagen, Hilden, Germany) and circulating cell-free DNA (cfDNA) was extracted from 8-10 ml of peripheral blood or pleural effusion using QIAamp Circulating Nucleic Acid kit, according to the manufacturer’s standard protocol (Qiagen, Hilden, Germany). Tissue genomic DNA was sheared into fragments (200~400 bp) using Covaris M220 (Covaris, Inc., Woburn, MA, USA). Fragmented genomic DNA and cfDNA were experienced with end repairing, A-tailing, and adaptor ligation and then was amplified by polymerase chain reaction (PCR) and purified. Fragments that were 200~400 bp in size were selected by beads (Agencourt AMPure XP Kit, Beckman Coulter, Brea, CA, USA), followed by hybridization with capture probes, hybrid selection with magnetic beads, PCR amplification and purified. Target capture was performed using six commercial panels. The panels consisting of 68, 168 and 520 cancer-related genes come from Burning Rock Biotech and the other three consisting of 14, 139 and 425 cancer-related genes come from Nanjing Geneseeq Technology Inc. The quality and the size of the fragments were assessed by high sensitivity DNA kit using Bioanalyzer 2100 (Agilent Technologies, CA, USA). For Burning Rock Biotech, the target-enriched libraries were sequenced on Nextseq 500 (Illumina, Inc., CA, USA) with paired-end reads and average sequencing depth of 1,000× for tissue and pleural effusion samples. For Nanjing Geneseeq Technology Inc., libraries were sequenced on HiSeq4000 (Illumina, Inc., CA, USA) with paired-end reads and average sequencing depth of 1,000× for tissue samples, 5,000× for plasma and pleural effusion samples.

***Sequence data analysis***

There is a little difference between the two companies about the principle of sequence data analysis. Hence, we described separately below.

For Burning Rock Biotech, sequence data were mapped to the reference human genome (hg19) using Burrows-Wheeler Aligner version 0.7.10. Local alignment optimization, duplication marking and variant calling were performed using Genome Analysis Tool Kit version 3.2, and VarScan version 2.4.3. Tissue and pleural effusion samples were compared against their own white blood cell control to identify somatic variants. Variants were filtered using the VarScan fpfilter pipeline, loci with depth less than 100 were filtered out. Base calling in pleural effusion and tissue samples required at least 8 supporting reads for single nucleotide variations (SNVs) and 5 supporting reads for insertion-deletion variations (Indels). Variants with population frequency over 0.1% in the ExAC, 1000 Genomes, dbSNP or ESP6500SI-V2 databases were grouped as single nucleotide polymorphisms (SNPs) and excluded from further analysis. Remaining variants were annotated with ANNOVAR (2016-02-01 release) and SnpEff version 3.6. Analysis of structural variations (SVs) was performed using Factera version 1.4.3. Copy number variations (CNVs) were analyzed based on the depth of coverage data of capture intervals. Coverage data were corrected against sequencing bias resulting from GC content and probe design. The average coverage of all captured regions was used to normalize the coverage of different samples to comparable scales. Copy number was calculated based on the ratio between the depth of coverage in tumor samples and average coverage of an adequate number (n>50) of samples without CNVs as references per capture interval. CNV is called if the coverage data of the gene region was quantitatively and statistically significant from its reference control. The limit of detection for CNVs is 1.5 for copy number deletion and 2.64 for copy number amplifications.

For Nanjing Geneseeq Technology Inc., sequencing data were demultiplexed by bcl2fastq (v2.19) and trimmomatic was used for FASTQ file quality control, leading/trailing low quality or N bases were removed. Qualified reads were then mapped to reference human genome (hg19) using Burrows-Wheeler Aligner. PCR duplicates were removed by Picard (Broad Institute, MA, USA) after local realignment around known Indels and base quality recalibration using Genome Analysis Toolkit (GATK 3.4.0). SNV and small Indels were called by VarScan2 and HaplotypeCaller/UnifiedGenotyper in GATK. Common SNPs were removed using dbSNP and the 1,000 Genome data sets. Germline mutations were filtered out by comparing them to the white blood cell controls. A mutation was called when the mutation allele frequency (MAF) cutoff was ≥ 0.5% for tissue and 0.1% for liquid biopsy samples, and a minimum of 5 and 3 unique mutant reads on different strands with good quality scores, respectively. All SNVs/indels were annotated with ANNOVAR, and each SNV/indel was manually checked on the Integrative Genomics Viewer (IGV). Gene fusions were identified by FACTERA and CNVs were analyzed with ADTEx, with default parameters. The log2 ratio cut-off for copy number gain was defined as 2.0 for tissue and 1.6 for cfDNA samples. A log2 ratio cut-off of 0.6 was used for copy number loss detection in all sample types.

**Supplementary Table 1.** The sequencing coverage and quality statistics of NGS for each sample.

| Sample ID | Sample type | Laboratory | NGS panel | Total number of sequenced reads | Total number of uniquely mapped non-duplicate reads | Total number of covered targeted bases | Median coverage (and range)per targeted base | Percentage of targeted base with coverage ≥200 (%) |
| --- | --- | --- | --- | --- | --- | --- | --- | --- |
| #01 | tissue | Nanjing Geneseeq Technology Inc | 139-panel | 1876118 | 1124183 | 126484108 | 1079(0-7575) | 99.35 |
| #02 | tissue | Burning Rock Biotech | 168-panel | 5678870 | 3181333 | 715396650 | 2193 (64-7585) | 99.70 |
| #05 | plasma | Nanjing Geneseeq Technology Inc | 425-panel | 44773643 | 17162286 | 3348082913 | 2306(0-8003) | 99.95 |
| #07 | plasma | Nanjing Geneseeq Technology Inc | 139-panel | 3091536 | 1311117 | 185897343 | 1773(0-7288) | 99.64 |
| #08 | tissue | Burning Rock Biotech | 68-panel | 7779904 | 2571656 | 811497450 | 1765 (11-4636) | 98.60 |
| #11 | pleural effusion | Nanjing Geneseeq Technology Inc | 425-panel | 56072188 | 23839132 | 4615791807 | 3010(0-8010) | 99.89 |
| #14 | tissue | Nanjing Geneseeq Technology Inc | 14-panel | 2834138 | 1279844 | 360000000 | 5604(1-22979) | 91.98 |
| #16 | tissue | Burning Rock Biotech | 167-panel | 9631802 | 5875399 | 995002045 | 3435(1-14292) | 97.60 |
| #20 | pleural effusion | Burning Rock Biotech | 520-panel | 32110482 | 17777490 | 3500671050 | 1546 (12-5322) | 98.90 |

NGS，next-generation sequencing

**Supplementary Table 2**. ORR, BOR, and DCR (ITT population).

|  | **Overall** | **PD-L1+** | **PD-L1-** | **ex20ins** | **G719X, etc.** | **BM** | **No BM** | **LM** | **No LM** |
| --- | --- | --- | --- | --- | --- | --- | --- | --- | --- |
|  | (N=21) | (n=7 ) | (n=8 ) | (n=12 ) | (n=9 ) | (n =9 ) | (n=12) | (n=4 ) | (n=17) |
| **BOR** |  |  |  |  |  |  |  |  |  |
| **CR** | 0 | 0 | 0 | 0 | 0 | 0 | 0 | 0 | 0 |
| **PR** | 8 (38.1) | 3 (42.9) | 3 (37.5) | 5 (41.7) | 3 (33.3) | 3 (33.3) | 5 (41.7) | 1 (25.0) | 7 (41.2) |
| **SD** | 10 (47.6) | 3 (42.9) | 4 (50.0) | 6 (50.0) | 4 (44.4) | 3 (33.3) | 7 (58.3) | 1 (25.0) | 9 (52.9) |
| **PD** | 3 (14.3) | 1 (14.2) | 1 (12.5) | 1 (8.3) | 2 (22.2) | 3 (33.3) | 0 | 2 (50.0) | 1 (5.9) |
| **ORR** | 8 (38.1) | 3 (42.9) | 3 (37.5) | 5 (41.7) | 3 (33.3) | 3 (33.3) | 5 (41.7) | 1 (25.0) | 7 (41.2) |
| **95% CI** | 18.1-61.6 | 9.9-81.6 | 8.5-75.5 | 15.2-72.3 | 7.5-70.1 | 7.5-70.1 | 15.2-72.3 | 0.6-80.6 | 18.4-67.1 |
| **DCR** | 18 (85.7) | 6 (85.7) | 7 (87.5) | 11 (91.7) | 7 (77.8) | 6 (66.7) | 12 (100.0) | 2 (50.0) | 16 (94.1) |
| **95% CI** | 63.7-97.0 | 42.1-99.6 | 47.3-99.7 | 61.5-99.8 | 40.0-97.2 | 29.9-92.5 | 73.5-100.0 | 6.8-93.2 | 71.3-99.9 |

Data are n (%) unless otherwise stated. Subgroups indicated in columns are not mutually exclusive.

BOR, best overall response; BM, brain metastases; CI, confidence interval; CR, complete response; DCR, disease control rate; LM, liver metastases; ORR, objective response rate; PD, progressive disease; PR, partial response; SD, stable disease
